# Plastid thioredoxins: a “one-for-all” redox-signaling system in plants

**DOI:** 10.3389/fpls.2013.00463

**Published:** 2013-11-21

**Authors:** Antonio J. Serrato, Juan Fernández-Trijueque, Juan-de-Dios Barajas-López, Ana Chueca, Mariam Sahrawy

**Affiliations:** ^1^Department of Biochemistry, Cell and Molecular Biology of Plants, Estación Experimental del Zaidïn, Consejo Superior de Investigaciones CientïficasGranada, Spain; ^2^Department of Molecular Plant Biology, University of TurkuTurku, Finland

**Keywords:** thioredoxin, redox signaling, photosynthesis, oxidative stress, carbon metabolism

## Abstract

The sessile nature of plants forces them to face an ever-changing environment instead of escape from hostile conditions as animals do. In order to overcome this survival challenge, a fine monitoring and controlling of the status of the photosynthetic electron transport chain and the general metabolism is vital for these organisms. Frequently, evolutionary plant adaptation has consisted in the appearance of multigenic families, comprising an array of enzymes, structural components, or sensing, and signaling elements, in numerous occasions with highly conserved primary sequences that sometimes make it difficult to discern between redundancy and specificity among the members of a same family. However, all this gene diversity is aimed to sort environment-derived plant signals to efficiently channel the external incoming information inducing a right physiological answer. Oxygenic photosynthesis is a powerful source of reactive oxygen species (ROS), molecules with a dual oxidative/signaling nature. In response to ROS, one of the most frequent post-translational modifications occurring in redox signaling proteins is the formation of disulfide bridges (from Cys oxidation). This review is focused on the role of plastid thioredoxins (pTRXs), proteins containing two Cys in their active site and largely known as part of the plant redox-signaling network. Several pTRXs types have been described so far, namely, TRX *f*, *m*, *x*, *y*, and *z*. In recent years, improvements in proteomic techniques and the study of loss-of-function mutants have enabled us to grasp the importance of TRXs for the plastid physiology. We will analyze the specific signaling function of each TRX type and discuss about the emerging role in non-photosynthetic plastids of these redox switchers.

## INTRODUCTION

Plant H_2_O photolysis provides electrons (and protons) to feed the photosynthetic electron transport chain (PETC) to allow NADPH and ATP synthesis for CO_2_ fixation. During the process, O_2_ and O_2_-derived by-products, called reactive oxygen species (ROS), are also released. However, ROS molecules (^1^O_2_, $O_{2}^{-}$ , H_2_O_2_, and ^•^OH) are even more oxidant than O_2_ itself (Noctor and Foyer, 1998). ROS-exposed cellular components (proteins, lipids, polysaccharides, and DNA) can be damaged, especially under environmental conditions leading to oxidative stress. Recent works also point to NO as an emerging oxidative compound (Lamotte et al., 2005; Grun et al., 2006; Neill et al., 2008a,b; Wilson et al., 2008) The NO-derived species are called reactive nitrogen species (RNS) and *S*-nitrosylation is the post-translational change they promote. ROS and RNS levels are controlled by thiol peroxidase-like plastid peroxiredoxins (PRX). Nevertheless, despite their toxicity at high concentrations, ROS and RNS play an important role as central signaling molecules (Laloi et al., 2004, 2007; Bellin et al., 2013).

Thiol groups (-SH) of cysteine residues are susceptible to ROS oxidation, provoking post-translational changes leading to enzyme inactivation. Upon oxidation, new chemical species can be generated from -SH, which can be gradually oxidized to sulphenic (-SOH), sulphinic (-SOOH), and sulphonic (-SOOOH) acids, the latter being an irreversible oxidation state. When a -SOH group is in the proximity of an -SH group, a disulphide bridge can form (-S-S-). This chemical reaction is of great biological relevance because thiol/disulphide inter-conversion operates as a molecular on/off switch for redox-regulated enzymes (Couturier et al., 2013). Plants, like other organisms, have developed sensing systems to monitor and maintain optimal redox conditions to avoid metabolic collapse. These sensing and signaling mechanisms (König et al., 2012) could be considered to be true “redox eyes”.

Glutathione (GSH) and ascorbate (Asc), the most abundant antioxidant compounds in plant cells (Noctor and Foyer, 1998), are considered to be unspecific reducer molecules because of their small molecular masses. However, plants have complex enzymatic antioxidant systems composed of thioredoxins (TRX) and glutaredoxins (GRX), known under the name of redoxins (RX). On the contrary to GSH and Asc, surface topology of RX allows specific target interactions (Wangensteen et al., 2001; Barranco-Medina et al., 2009; Arsova et al., 2010). Among the first RX targets identified at the beginning of proteomic era were PRX (Baier and Dietz, 1999a,b, 2005; Dietz et al., 2006), which are involved in ROS/RNS signaling (König et al., 2012) and, as mentioned above, key ROS detoxifying enzymes. RX are highly diversified in plants and display a conserved tertiary structure (TRX folding) holding one or two Cys at their active sites. Useful reviews are available on plant GRX and their cross-talk with TRX (Rouhier et al., 2006; Xing et al., 2006; Meyer et al., 2009, 2012; Zaffagnini et al., 2012). TRX are low-redox-potential proteins (<-270 mV) of approximately 10–12 kDa with the conserved active site WC(G/P)PC (classical TRX). The Cys residues of the TRX active site switch from a reduced (sulphydryl groups) to an oxidized form (disulphide bridge) as part of the enzymatic mechanism resulting in the reduction of a target protein.

Plants are sessile eukaryotic photosynthetic organisms that have colonized multitude of terrestrial environments with fluctuating light intensities, water availability, temperature variations, and other environmental factors continuously challenging plant life. Success of this adaptation lies partially in the versatile redox signaling and regulation exerted by TRX (König et al., 2012). With the arrival of the genomics era and massive sequencing projects, many plant species have been already sequenced (e.g., *Arabidopsis thaliana* and *Oryza sativa*). The knowledge of full genomes offered the possibility of discovering tissue-specific or faintly expressed TRX, elusive before genomics. At present, the number of TRX, TRX-like proteins, or proteins with TRX-domains in *Arabidopsis* have risen to 44 members (Meyer et al., 2012), many of them without any assigned function. TRX are classified, according to their subcellular location and sequence similarity, into 15 subgroups (Meyer et al., 2012). While classical TRX *h* and *o *are located in cytosol/nucleus and mitochondria, respectively, five typical TRX exist in plastids, namely, TRX *f*, *m*, *x*, *y*, and *z*. TRX receive electrons from two compartment-specific and well-defined systems: the ferredoxin-thioredoxin system (FTS), which reduces plastid TRX with electrons coming from ferredoxin through the action of ferredoxin-thioredoxin reductase (FTR); and the NADP-thioredoxin system (NTS), involving the NADPH-thioredoxin reductase (NTR) to furnish electrons from NADPH to TRX *h* and *o*. Apart from FTS and NTS, NTRC is a bi-modular protein with NTR and TRX domains reported in 2004 (Serrato et al., 2004). Although NTRC is located in plastids, it is nevertheless reduced by NADPH (Spínola et al., 2008; Pérez-Ruiz and Cejudo, 2009) and behaves as a condensed NTS system important for the response to abiotic and oxidative stress (Serrato et al., 2004; Pérez-Ruiz et al., 2006). Besides the antioxidant role, NTRC functions related to carbon metabolism have been recently proposed (Michalska et al., 2009).

Phylogenetic studies on plastid TRX and sequence comparisons have demonstrated that while TRX *m*, *x*, *y*, and *z* are of prokaryotic origin (Sahrawy et al., 1996; Arsova et al., 2010), TRX *f* is closely related to eukaryotic TRX (Sahrawy et al., 1996; Issakidis-Bourguet et al., 2001). It seems reasonable that TRX diversification reflects the complexity of the plastid redox network and the extent of their role played in plant physiology. In recent years, due mainly to the availability of collections of mutant lines, many studies on plastid TRX have focused on the model plant *A. thaliana*. In this species, two *f*, four *m*, two *y*, and one *x* and *z* TRX isoforms have been described. This multiplicity has raised the question of functional redundancy or a specific role for each isoform. In this sense, Issakidis-Bourguet et al. (2001) showed that chloroplast TRX *f*, *m*, and *x* are differentially able to compensate for TRX deficiency in yeast. Since the discovery some decades ago of the preferential activation of chloroplast fructose-1,6-bisphosphatase (FBPase) by TRX *f* and NADP-malate dehydrogenase (MDH) by TRX *m* (Schürmann et al., 1981), linking carbon fixation and TRX-mediated activation, many other essential plastid processes such as PETC, oxidative-stress response, starch metabolism, nitrogen metabolism, lipid biosynthesis, protein folding, protein import, translation, or chaperone activity (Balmer et al., 2004; Buchanan and Balmer, 2005; Balsera et al., 2010; Chibani et al., 2010; Sanz-Barrio et al., 2012) have been reported to be under the redox regulation exerted by TRXs. Moreover, initially confined to chloroplasts, growing evidence points to new physiological functions in roots and other heterotrophic organs (Barajas-López et al., 2007; Traverso et al., 2008; Benitez-Alfonso et al., 2009; Fernández-Trijueque et al., 2012).

## TRX *f* AND *m* ARE PHOTOSYNTHESIS-RELATED ENZYMES, BUT NOT EXCLUSIVELY SO

Discerning between functional specificity and redundancy among components of multigenic families proves difficult. Single loss-of-function lines are frequently phenotypically undistinguishable from wild-type plants. To address this question, one possibility would entail obtaining double, triple, or even quadruple loss-of-function mutants. Nevertheless, this approach is time consuming and, in the case of TRX, can be complex because of cross-talks with the GRX family. Some authors have evidenced this cross-talk by inhibiting GSH synthesis (Reichheld et al., 2007). Expression patterns (abundance and tissue location), protein topologies (determining electrostatic and/or hydrophobic interactions), redox potentials, and post-translational modifications are distinctive features that would address the specificity for each pTRX toward a particular target in a specific cell type. Described long before other pTRXs, greater information has been compiled on TRX *f* and *m* than on the *x*, *y*, or *z *isoforms. Most recent works have offered further insight into the specific role of TRX *f* and *m* in photosynthesis, carbohydrate metabolism, NADPH synthesis, response to abiotic stress and, notably, putative new functions in heterotrophic organs.

## REDOX SIGNALING IN PHOTOSYNTHESIS REGULATION

**FIGURE 1:**
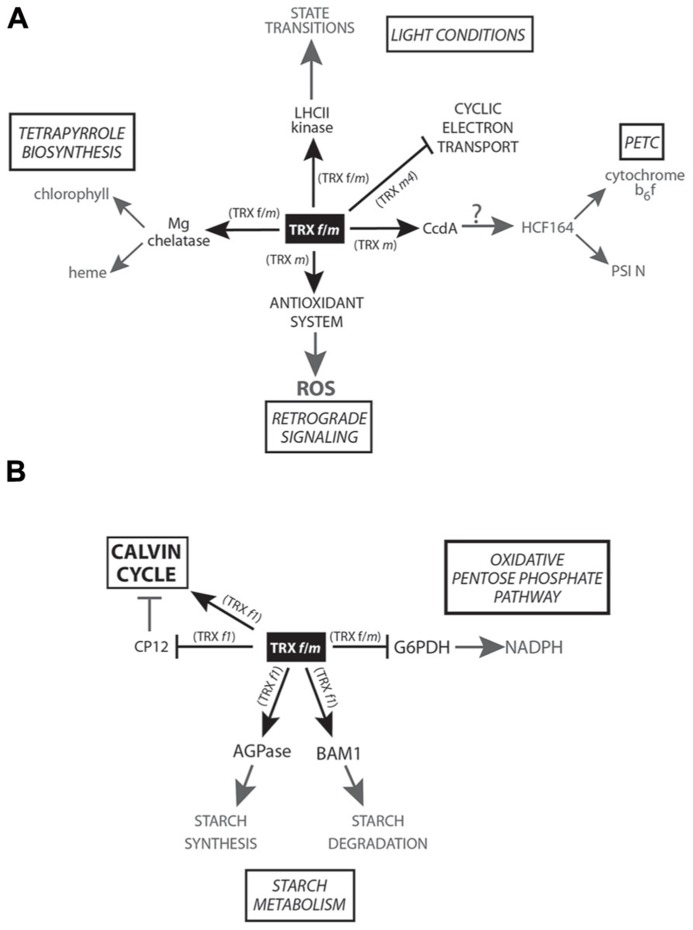
**Scheme of the TRX *f* and *m*-mediated redox signaling in plastids. (A)** Main physiological processes related to photosynthesis and retrograde signaling controlled by TRX *f* and/or *m *are shown in the figure. **(B)** Regulation of carbon metabolism carried out by TRX *f* and/or *m*.

A large cluster of genes involved in light-harvesting reactions of photosynthesis genes coding for LHCA and LHCB (Light Harvesting Complex) proteins, protoporphyrin IX Mg chelatase, and several proteins of the photosystem I and II reaction centers (PSI and PSII, respectively) are found to be under clock control (Harmer et al., 2000). ATPase activity of Mg chelatase CHLI subunit, from the tetrapyrrole biosynthesis pathway, is activated *in vitro *by *Pissum sativum *TRX *f* (Luo et al., 2012). *In vivo* experiments with TRX *f* virus-induced gene-silenced plants produced no phenotype changes in the treated plants, suggesting that low levels of TRX *f *could be compensated for by the *m*-type isoform (Balmer et al., 2003; Luo et al., 2012). Nevertheless, in the same work, the silencing of pea TRX *f*/*m* induced a pale-green phenotype and ROS accumulation. The authors suggest two possible types of TRX-mediated regulations for the tetrapyrrole biosynthesis pathway: one being transcriptional regulation through plastid-mediated retrograde signaling; and another being an indirect result of the lower Mg chelatase (interacting *in vitro* with TRX *f*) activity due to lower TRX *f*/*m* activity (**Figure 1A**).

It is known that carbon metabolism is concomitantly under this circadian rhythm. Therefore, it seems reasonable, given the tight relationship with photosynthesis and carbon fixation, that pTRX genes should have a similar transcriptional regulation in order to optimize these physiological processes. Surprisingly, only two (TRX *f2* and *m2*) out of the six TRX *f* and *m* of *A. thaliana *and the pea TRX *f* and *m1 *follow a circadian rhythm (Barajas-López et al., 2011). The rest of TRXs *f* and *m*, with the exception of TRX *m3*, are directly induced by light. This light-independent gene expression could correspond to a more functional specificity of TRX *m3* (Benitez-Alfonso et al., 2009), clustered away in phylogenetic trees based on protein-sequence comparisons (Arsova et al., 2010).

Regarding photosynthesis regulation (**Figure 1A**), TRX is involved in state transitions (Rintamäki et al., 2000). Both TRX* f* and *m* are able to *in vivo* inactivate LHC kinase in response to high light intensities, controlling the relocation of LHC between PSI and PSII under this condition. More recently, in 2006, TRX *m* and *f* were found to transfer reducing equivalents (the *m-*type isoform being more efficient) to HCF164, a thylakoid-membrane-spanning protein with two thioredoxin-like domains participating in the assembly of cytochrome *b*_6_f (Lennartz et al., 2001), mediating electron transport between PSII and PSI, and the reduction of the photosynthetic protein PSI-N both *in vitro* and in isolated thylakoids (Motohashi and Hisabori, 2006). HCF164 reduction might involve CcdA, a thylakoidal protein from bacterial origin targeted by TRX *m* (Motohashi and Hisabori, 2010). However, this hypothesis is only based on indirect results obtained from *in vitro* subcellular localization experiments and phenotype similarities between the *ccda* and *hcf164* *Arabidopsis* mutants. Further *in vitro* and/or *in vivo* experimental approaches will be necessary to corroborate this putative interaction and its regulation. Very recently, the analysis of the photosynthetic parameters of an *A. thaliana* loss-of-function mutant has allowed to uncover a direct photosynthesis control carried out by TRX *m4 *(Courteille et al., 2013). According to the authors, this TRX *m *isoform would play an important role in regulating photosynthetic alternative electron pathways in *A. thaliana* and *Nicotiana tabacum* chloroplasts, acting as a repressor of the cyclic electron flow (CEF) involved in preserving a proper ATP/NADPH balance. Although this was the first specific function assigned to TRX *m4*, the physiological significance of this regulation still remains to be clarified. In this sense, the photosynthesis regulation of TRX *m* has been shown in rice, when the repression of OsTRX *m* level is responsible for a greater reduction, compared to wild-type plants, in the photosynthetic efficiency under high-irradiance treatments together with other impairments such as thylakoidal ultrastructural changes and a reduced chlorophyll and pigment content (pale-green leaves; Chi et al., 2008).

Until now, pTRX regulation of photosynthesis has been *in vivo* studied in C3 plants. In C4 photosynthesis, the redox state of the bundle sheat cells and mesophyll cells are expected to have very different redox status due to the spatial separation of the photosynthetic process. This C4-photosynthesis peculiarity could be influencing the relative expression pattern of the different pTRX isoforms in bundle sheat and mesophyll cells and how the photosynthesis is redox controlled in C4 plants. We hope that this intriguing topic could be addressed in a near future.

## CONTROL OF CARBOHYDRATE METABOLISM IN PLASTIDS

Sugars are photosynthetic products and, recently, it has been reported that these molecules (together with thiol status in leaves) are regulating the expression of *PsTRXf* and *m1* genes in pea plants (Barajas-López et al., 2012). This regulation is mainly exerted by glucose and sucrose and might involve the transcription factor *PsDOF7*, able to bind to a pea DOF motif present in *PsTRX f* and *m1* promoters (Barajas-López et al., 2012).

Today, the redox regulation of all Calvin cycle (CC) enzymes exerted by pTRXs is widely acknowledged (Lindahl and Kieselbach, 2009; Meyer et al., 2012). Concerning redox regulation, one of the most intensely studied CC enzymes has been the chloroplast fructose-1,6-bisphosphatase (cFBPase), whose redox activation mechanism is a classical model in enzyme post-translational regulation (Jacquot et al., 1995; Chen and Xu, 1996; Hermoso et al., 1996; Jacquot et al., 1997; López Jaramillo et al., 1997; Chiadmi et al., 1999; Wangensteen et al., 2001; Cazalis et al., 2004). Nevertheless, most of the interaction evidence comes from *in vitro* studies. In immunocytolocalization experiments with pea chloroplasts, TRX *f *and *m* have been found to be non-randomly distributed with respect to some CC-analyzed enzymes, NADP-dependent malate dehydrogenase (NADP-MDH), heat-shock protein 70 (Hsp70), and ATP synthase (Anderson et al., 2008). Based on pTRXs co-localization with non-light activated enzymes, the authors proposed a secondary function for pTRXs as protein linkers facilitating enzyme interactions and/or substrate channeling. Additionally, it has been suggested that mechanisms by means pTRXs would exert a fine-tuned modulation of enzyme activities based on short-lived interactions, not only involving a simple binary on/off mechanism (König et al., 2012).

Apart from the pTRX-mediated activation of CC target enzymes, an additional and indirect activation mode involving CP12, a small chloroplast protein containing four redox-active Cys, was described in the 1990s (Wedel et al., 1997). In its oxidized state, CP12 forms an inhibited complex (**Figure 1B**) with the CC enzymes NADPH-glyceraldehyde-3-phosphate dehydrogenase (GAPDH) and phosphoribulokinase (PRK), fully restored upon pTRXs reduction (Marri et al., 2009). In plant cells, pTRXs/CP12 system broadens the redox regulatory complexity, providing a faster activation of CC oxidized enzymes and pointing to TRX *f1* as the physiological reducer of GAPDH/CP12/PRK complexes in *A. thaliana*. Further *in vivo* studies have highlighted the importance of CP12 in carbon partitioning and growth in tobacco plants, leading to proposal of functions other than the single formation of PRK and GAPDH complexes (Howard et al., 2011).

Plastid synchronization of the starch synthesis/degradation processes is crucial for plant growth and development. ADP-glucose pyrophosphorylase (AGPase) is a key redox-activated enzyme for starch biosynthesis in plastids (Fu et al., 1998; Ballicora et al., 2000). Very recently, in *Arabidopsis* leaves, the role of TRX *f1* as AGPase activator (**Figure 1B**) during light period has been evidenced (Thormählen et al., 2013). In a loss-of-function *trx f1* mutant, a lower redox activation of AGPase and a decrease in the starch:sucrose ratio have been detected during the day. AmongTRX *f1*, *m1*, *x*, and *y1*, AGPase is more efficiently *in vitro *activated by TRX *f1 *(followed by TRX *m1*). These authors hypothesized that AGPase could be activated by TRX *f1* during the light period while NTRC would be the activating enzyme in the dark. However, other authors have reported contradictory experimental results and contend that redox modulation is of minor importance for AGPase activity in response to light (Li et al., 2012). Nevertheless, further experiments will be necessary to clarify this important point.

Surprisingly, not only starch synthesis is TRX *f1* controlled but also degradation through the redox-activated enzyme BAM1 (**Figure 1B**), an *A. thaliana* β-amylase controlling stomata opening (Valerio et al., 2011). Although TRX *f1* activates both starch synthesis and degradation during the day, these processes take place in different cell types (mesophyll cells and guard cells, respectively). However, light degradation in mesophyll tissue is activated during osmotic-stress situations that trigger BAM1 induction. *BAM1* is also expressed in *Arabidopsis* roots, where NTRC (proved to be less efficient than TRX *f *in *in vitro* assays) is the putative activating enzyme (Valerio et al., 2011). Besides the presumed role of NTRC as a redox alternative activator under dark conditions and/or in non-photosynthetic organs, it is feasible that classical pTRXs (the specifically expressed in non-photosynthetic organs) could be activated by the heterotrophic ferredoxin NADP reductases (FNR) isoforms and NADPH (Hanke et al., 2004; Balmer et al., 2006; Barajas-López et al., 2007; Bohrer et al., 2012; Fernández-Trijueque et al., 2012). Despite that some works have focused on the study of pTRX-mediated redox regulation in non-photosynthetic organs, much effort still needs to be done in order to ascertain the true extent of pTRXs in heterotrophic tissues.

## OXIDATIVE ACTIVATION OF GLUCOSE-6-PHOSPHATE DEHYDROGENASE

Glucose-6-phosphate dehydrogenase (G6PDH) catalyzes the first committed step of the oxidative pentose phosphate pathway (OPPP), a major source of NADPH for plant heterotrophic cells as well as for photosynthetic tissues during the night period. Six genes coding for G6PDH have been identified in *Arabidopsis*, four predicted to code for plastid isoforms (Wakao and Benning, 2005). The higher number of G6PDH identified in plastids points to the significance of OPPP taking place in this subcellular compartment. *In vitro* assays have shown the reductive inactivation by DTT, a common feature of at least three out of the four plastidial enzymes (AtG6PDH1, AtG6PDH2, and AtG6PDH3) and not shared with the cytosolic isoforms. *AtG6PDH1* is expressed mostly in photosynthetic tissues while *AtG6PDH2* and *AtG6PDH3* transcripts are accumulated predominantly in roots (Wakao and Benning, 2005). Although a specific TRX *m*-mediated G6PDH inactivation (**Figure 1B**) has been previously reported (Wenderoth et al., 1997), Née et al. (2009) have demonstrated that *A. thaliana *TRX *f1* regulate AtG6PDH1 activity *in vitro *as efficiently as TRX *m1* or *m4*. Nevertheless, these *in vitro *results must be carefully interpreted and need to be supported by complementary *in vivo* interaction approaches or by determining the *in vivo* redox state of G6PDH1 in a TRX *f1* loss-of-function mutant. In addition, it would be helpful to know whether pTRXs also regulate root-expressed AtG6PDH2 and AtG6PDH3 in order to elucidate the role of pTRXs in OPPP control and NADPH synthesis. It is tempting to conclude that some of the pTRXs could control their own redox status through the activation/inactivation of OPPP in heterotrophic organs.

## ROS HOMEOSTASIS IN ROOTS

*TRX m3* transcripts are one of the least abundant pTRXs mRNA in leaves, while higher root-transcript levels are comparable to those of *TRX m2*, *m4*, and *x *(Bohrer et al., 2012). Nevertheless, until now, the published results highlight the importance of TRX *m3* for root ROS homeostasis (Benitez-Alfonso et al., 2009; Benitez-Alfonso and Jackson, 2009). The results shown in this work localize TRX *m3* in root and shoot meristem plastids, this isoform being important for callose deposition and plasmodesmal transport, as well as for arresting the growth of TRX *m3 *loss-of-function seedlings (*gat1* mutant). It is quite surprising, however, that, given the redundancy of ROS detoxifying mechanisms in plant cells and the existing interplay between TRX and GRX signaling pathways, in vital plant meristems, the ROS content was not buffered by other TRX isoforms or GRX members also expressed in roots (Bohrer et al., 2012; Meyer et al., 2012). In fact, other authors hold that the lethality due to TRX *m3* inactivation needs to be firmly established (Reichheld et al., 2010).

Although some studies have experimentally proved the presence of *Pisum sativum *TRX *f* and *m *isoforms in roots (Barajas-López et al., 2007) and the response of TRX *m* to NaCl-induced stress in root pea seedlings (Fernández-Trijueque et al., 2012) there is no information about the precise tissue expression in this organ (excepting *TRX m3*). According to the online available microarray data from “*Arabidopsis* eFP Browser” (), *TRX m2* would be the most abundantly expressed gene in roots, principally in procambium cells. In contrast, *TRX m1* would be pericycle and phloem specific while *TRX m4* could be mostly expressed in cortex and procambium tissues. The analysis of the data suggests a poor expression in *A. thaliana *of both *TRX f *genes in roots compared to the other *TRX m* genes. Nonetheless, although these data may help to have an approximate idea about the pTRX accumulation in root tissues, we must taking into account that putative further post-transcriptional control could alter the final protein-expression pattern.

## TRX *x* AND *y*: THE SUBSET OF pTRXs SPECIALIZED IN RESPONDING TO OXIDATIVE STRESS

**FIGURE 2:**
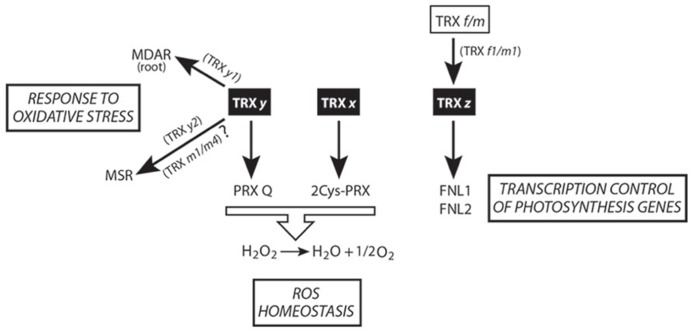
**Scheme of TRX *x*, *y*, and *z*-mediated redox signaling in plastids.** The central physiological processes regulated by these isoforms are shown.

The first reports describing TRX *x* and *y* as members of the plant pTRXs appeared in 2003 and 2004, respectively (Collin et al., 2003; Collin et al., 2004). It bears noting that TRX *x*, *y*, and *z* have higher redox potentials (>-340 mV) than do TRX *f* and *m* (<-350 mV; Collin et al., 2003; Collin et al., 2004; Chibani et al., 2011). As mentioned above, one of the key TRX features determining their functional specificity is redox potential. This biochemical characteristic confers these proteins a poor capacity to activate carbon-metabolism enzymes such as FBPase and MDH. On the contrary, *x*- and *y*-type TRX can efficiently activate plastid 2-Cys PRX and PRX Q, respectively (**Figure 2**). The absence of TRX *x* in the *A. thaliana* mutant *trxx* triggers protein carbonylation (stress marker) but does not affect photosynthesis or carbon fixation under long-day conditions (Pulido et al., 2010). Nevertheless, under continuous-light conditions, CO_2_ fixation is affected in *trxx*, suggesting that TRX *x *can undertake a more important role under non-optimal environmental conditions (Pulido et al., 2010).

Two *y*-type isoforms, TRX *y1* and *y2*, are present in *Arabidopsis*. These oxidoreductases do not activate FBPase and only partially MDH, being efficient reducers of PRX Q (Collin et al., 2004). In *Arabidopsis* plants, TRX *y1* is preferentially expressed in heterotrophic organs (e.g., roots and seeds), while TRX *y2* is a more photosynthesis-associated protein. Expression in heterotrophic organs of the *y1*-type implies the existence of a functional redox system, furnishing reducing power to these TRX. In this sense, Marchand and colleagues reported a list of TRX *y* targets in *Arabidopsis* roots, finding numerous proteins involved in detoxification and defense (**Figure 2**) like monodehydroascorbatereductase (MDAR; Marchand et al., 2010). Notably, although *y*-type TRX cannot activate FBPase, another of the root-found targets is a putative fructose bisphosphate aldolase, whose reaction product that serves as a substrate for cFBPase and that participates in plant responses to abiotic stress (Lu et al., 2012).

Recently, from the analysis of methionine sulfoxidereductase (MSR) capacity in different TRX loss-of-function mutants, TRX *y2 *has been proposed as the physiological electron donor of MSR (**Figure 2**; Laugier et al., 2013). However, according to the results shown in this work, overlapping functions of TRX *m1* and *m4* as MSR activators cannot be ruled out.

## TRX *z*, A REDOX REGULATOR OF THE PLASTID TRANSCRIPTION

Although a *Solanum lycopersicum* TRX* z *ortholog (CITRX) has been reported as an interacting cytosolic partner of the resistance protein Cf-9 (Rivas et al., 2004), in 2006 this protein was identified for the first time as a component of plastid transcriptionally active chromosomes (TACs) from mustard (*Sinapis alba*) and *Arabidopsis* (Pfalz et al., 2006) and, 4 years later, named as TRX *z *(Arsova et al., 2010) or TRX *p *(Meng et al., 2010) and designated as a new member of pTRXs. The lack of this protein affects transcription (**Figure 2**) of genes dependent on plastid-encoded RNA polymerase (PEP), essentially photosynthetic-related genes (class I; Arsova et al., 2010). Consequently, *A. thaliana trxz* has yellow leaves and lacks the ability of autotrophic growth while in *Nicotiana benthamiana *low TRX *z *protein levels induce a chlorotic phenotype (Arsova et al., 2010; Meng et al., 2010). Two fructokinase-like proteins (FLN1 and FNL2) reportedly interact *in planta* with TRX *z* in a thiol-dependent way. Recombinant FLN1 and FLN2 lack any sugar-phosphorylating activity, suggesting a regulatory rather than a metabolic function (Arsova et al., 2010). Several pieces of evidence, such as *in planta* interaction between FLNs and TRX *z*, a similar *Arabidopsis* leaf phenotype of the *trx z* and the FLN1 and FNL2 silenced mutants, and a reduced expression of PEP-dependent class I genes in both mutants suggest that TRX *z* and FLN1 and FLN2 might take part in a signaling pathway, regulating PEP activity in chloroplasts (Arsova et al., 2010). It is noteworthy that, in a parallel work, both TRX *z* and FNL1, together with other redox proteins, have been reported to take part of PEP complexes in mustard (Schröter et al., 2010). Curiously, the TRX *f*-target FBPase (Schürmann et al., 1981) is among the proteins found in mustard transcriptional complexes.

Biochemical assays with poplar TRX *z* have shown that this protein can be reduced by NTRB (Chibani et al., 2010), physiologically important in the case that TRX *z* is dually targeted to plastids and cytosol (Rivas et al., 2004). The activation of some peroxidases and MSR has led Chibani et al. (2011) to propose TRX *z* as an alternative electron donor to ROS-detoxifying enzymes. *Arabidopsis* TRX *z* is able to form dimers in its oxidized state, being monomerized upon reduction by DTT and, unlike TRX *x*, *y*, and *f1*, is the first pTRX not reduced by FTR (Bohrer et al., 2012). The higher redox potential of TRX *z* with respect to other pTRXs prompted to Bohrer and colleagues to conduct *in vitro* reduction assays by using other pTRXs. TRX *f1* and *m1* behaved as good TRX *z* reducers, being the first available case of TRX reduced by other TRX (**Figure 2**). However, additional *in vivo* experiments (as the determination of the reduction/oxidation TRX *z* state in loss-of-function pTRX mutants) need to be performed in order to corroborate these intriguing results.

## PLASTID CYSTATHIONINE β-SYNTHASE DOMAIN-CONTAINING PROTEINS REGULATE pTRXs ACTIVITY

In the literature, no activating-TRX protein has been reported prior to the work of Yoo et al. (2011), which demonstrated the activating role of plastid cystathionine β-synthase (CBS) domain-containing proteins (CDCPs) over FTS and NTS. CDCPs are members of a large superfamily of ubiquitous proteins able to bind to adenosine-containing ligands such as AMP, ATP, or *S*-adenosyl methionine (Yoo et al., 2011). In *A. thaliana* and rice, 34 and 59 CDCPs have been reported, respectively (Kushwaha et al., 2009). CDCPs are located in different subcellular compartments. Two of these proteins, CBSX1 and CBSX2, are located in plastids and are able to activate TRX *f*, *m*, *x*, and *y *(Yoo et al., 2011). The loss-of-function mutant *cbsx1* shows severe growth retardation while CBSX1 overexpressing plants are able to grow faster in free-sucrose medium and display a delayed senescence compared to wild-type plants, resembling transgenic plants overexpressing TRX *m *(Benitez-Alfonso et al., 2009). Notably, the authors have suggested that CBSX1 would regulate physiological processes in non-green tissues while CBSX2 would be a green-tissue specific protein, reinforcing the above-mentioned idea of the presence of a fully active FTS in heterotrophic organs.

## CONCLUDING REMARKS

Sometimes, phenotypic differences between TRX mutant lines and wild-type plants are subtle or even missing. However, in order to see whether novel isoforms have conferred adaptive advantages during evolution it would be necessary to perform population-dynamics studies of loss-of-function mutants grown under natural conditions. Although we know that this approach would be time-consuming and difficult to develop, it would give a definite answer to the perennial question of the functional specificity or redundancy of the members of the family of TRX. Additionally, to find a putative relationship between environment adaptation and diversification of plant pTRXs, it would be interesting to analyze whether there would be differences between the number of pTRX isoforms found in plant species living in extreme environments (e.g., deserts) compared with other species living in more stable environments (e.g., rain forests). It is quite probable that the diversification of the pTRXs also responds, at least in part, to the demand of a more complex redox signaling due to the appearance of new specialized organs (and plastid types), as roots and flowers, necessary for the successful land colonization. In roots and some flower tissues, and instead of chloroplasts, specialized non-green plastids are present. It would be logical to think that plants have reprogrammed or adapted the redox-signaling machinery already present in green plastids to redox regulate the light-independent processes occurring in non-green plastids. However, with the exception of a few works already mentioned in this review, there is very little information about the pTRX targets in heterotrophic organs and the light-independent processes they are redox regulating. Further comprehensive studies are still necessary to realize the extent of the redox-signaling role mediated by pTRX in the whole plant.

In our opinion, when we study multigenic families, we almost exclusively focus our attention on the gene coding sequences and on comparative analyses of the primary structures of the peptides they are coding for. However, regulatory sequences (DNA motifs) present in promoters are also a basic part of genes (and sometimes neglected). When revising TRX literature it is quite usual to find *in vitro* interaction experiments in which two or more pTRX isoforms are reported to have the same or similar affinity for a given target. For instance, *Arabidopsis* TRX *f2* isoform has been omitted from the *in vitro *interaction experiments of some works reasoning a high sequence similarity with TRX *f1*. We think that the diversification of the TRX family and other multigenic families could also respond to a plant strategy leading to a more efficient transcriptional regulation. The increase in the number of plant transcription factors and the complexity of the transcriptional machinery could have compromised gene regulation and, consequently, plant survival. One solution could have consisted in organizing regulatory DNA motifs in several promoters. These regulatory sequences could have co-evolved together with the coding regions following a gene duplication event. During evolution, both the promoter changes as the amino acid substitutions of the pTRXs could allow a precise and specific redox signaling in non-green plastid of the heterotrophic organs.

**FIGURE 3:**
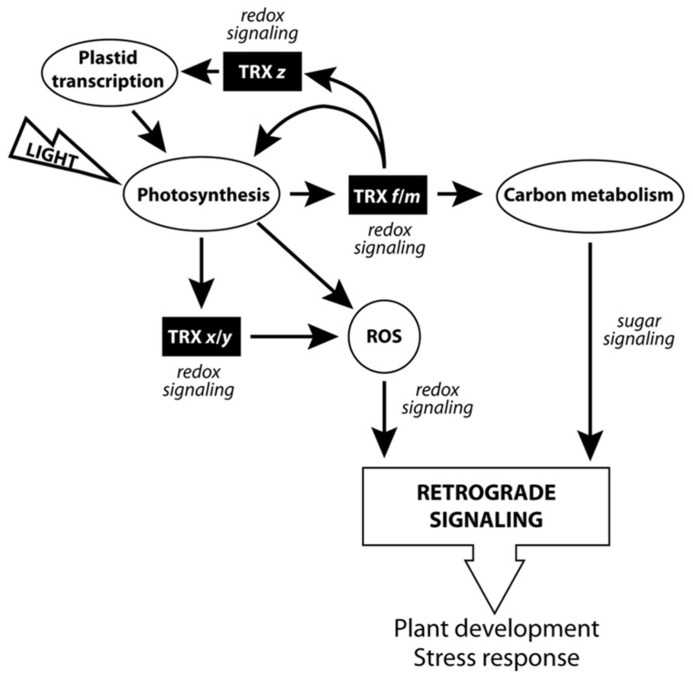
**Scheme of the pTRXs implication in the chloroplast signaling network**.

As we have pointed out, pTRXs redox proteins may regulate a large number of plant physiological processes, and compelling evidence points toward the existence of fully active pTRXs in heterotrophic tissues. According to the works cited in this review, three pTRX functional subsets can be inferred (**Figure 3**). The first subgroup, related to photosynthesis and carbon metabolism, would be composed of the TRXs *f* and *m* isoforms, coupling light and redox-signaling pathways (excluding the *Arabidopsis* TRX *m3* isoform, possibly developing a specific physiological role). In the second subcategory, we could find the *x*- and *y*-type TRXs, involved mostly in ROS detoxification and taking part of the complex redox-signaling network regulating plant development. The last subset, related to redox signaling and regulation of photosynthesis-related transcription in chloroplasts, would be composed of only one member, i.e., TRX *z*. Intriguingly, TRXs *f* and *m* are efficient TRX *z *reducers. Subsequently, it is tempting to conclude that *f*- and *m*-type isoforms, in addition to regulate the photosynthesis-related processes mentioned in this review, could also act as redox-signaling molecules linking photosynthesis and plastid transcription. In our opinion, the discovery of CDCPs as pTRX activity regulators should be taken into account in order to improve our knowledge of the external elements modulating the multilevel redox signaling mediated by pTRXs.

## Conflict of Interest Statement

The authors declare that the research was conducted in the absence of any commercial or financial relationships that could be construed as a potential conflict of interest.
